# Intestinal Ischemia Due to Methamphetamine Abuse in a Confused Elderly Patient: A Case Report

**DOI:** 10.7759/cureus.31192

**Published:** 2022-11-07

**Authors:** Emad Sherkawi, Sara A Aljaafari, Ziyad T AlGhannam, Fatimah A Alsafar

**Affiliations:** 1 General Surgery, Johns Hopkins Aramco Healthcare, Dhahran, SAU; 2 Medicine, Imam Abdulrahman Bin Faisal University, Khobar, SAU; 3 Medicine, King Faisal University, Al-Ahsa, SAU

**Keywords:** elderly population, drug addiction, drug abuse, non-occlusive bowel ischemia, methamphetamine

## Abstract

Bowel ischemia is a critical entity that mandates an early and proper diagnosis. Causes of bowel ischemia are numerous, yet, identifying and treating the exact cause is challenging. Methamphetamine-induced bowel ischemia is rare but of clinical significance due to its high disease burden.

We describe a case of a 67-year-old man who presented with colicky abdominal pain shortly after methamphetamine intake. CT angiography was done and failed to show occlusive causes, which pointed to a non-occlusive cause of bowel ischemia. The patient was hemodynamically stable on admission. Diagnostic laparoscopy was converted to laparotomy; segmental gangrene of both the small and large bowels was found. Resection of the ischemic part was done. The patient improved and was hemodynamically stable postoperatively.

In conclusion, a holistic approach to patient history and physical examination can grab attention to unusual pathologies that lead to early intervention and fastened life-saving measures. Questions about stimulant drug use are crucial not only in younger patients but in older patients as well. In this case, we highlight the surgical, medical, and mental aspects of methamphetamine abuse in the elderly population.

## Introduction

Methamphetamine is one of the most common forms of amphetamine used illicitly. It is usually sold as a powder, or in crystal form (known as ‘ice’). The powder form is usually snorted or injected; the crystal methamphetamine is usually smoked or injected. The crystalline form is also suitable for vapor inhalation [1]. Methamphetamine is a psychostimulant drug that causes the release of central and peripheral monoamines, including dopamine, serotonin, and norepinephrine [2]. This psychoactive drug is neurotoxic and proven to cause cognitive deficits including a range of adverse impacts on attention, executive functions, information processing, episodic, visual, learning memory, language, and motor skills [3-5]. However, its production and abuse have continued to increase over the years [6,7]. There are well-documented cardiovascular and cerebrovascular pathologies of this drug. However, gastrointestinal pathology is less understood and only a few cases of bowel ischemia due to splanchnic vasoconstriction have been reported in the literature [8-12], and almost none in elderly patients. Here, we report the case of an elderly patient who presented with life-threatening bowel ischemia which was found to be related to methamphetamine abuse.

## Case presentation

A 67-year-old male patient presented to the emergency department with three days of acute upper abdominal colicky pain. The pain was noticed shortly within hours of methamphetamine intake. The pain was not associated with vomiting or altered bowel habit; there was no history of fever. Past medical history revealed coronary artery disease with stenting, hypertension, and heavy smoking. Psychiatric history revealed depression, psychosis, and illicit daily methamphetamine drug abuse. Regarding social history, the patient stated that he is divorced and is living alone. His surgical history was unremarkable.

On arrival at the emergency department, the patient was restless and confused. His vitals were as the following: temperature of 37.3◦C, pulse of 112 bpm, respiratory rate of 19, oxygen saturation of 100%, and blood pressure of 194/71 mmHg. The abdominal examination showed a distended upper abdomen with guarding but no rigidity and no rebound tenderness. Per rectum examination was unremarkable; it revealed an empty rectum with no mass and no blood.

During admission, laboratory investigations revealed elevated white blood cells 18.2 K/cumm (4.0 - 10.0 K/cumm), elevated lactic acid 6.8 mmol/L (0.7 - 2.1 mmol/L), elevated AST 88 IU/L (17 - 59 IU/L), troponin high sensitive 0.073 ng/mL (0.000 - 0.026 ng/mL), and elevated B-type natriuretic peptide 1824 pg/mL (<100 pg/mL) (Table 1).

**Table 1:** Lab values ALT: alanine aminotransferase; AST: aspartate aminotransferase; WBC: white blood cells; RBC: red blood cells; MCV: mean corpuscular volume; MCH: mean corpuscular hemoglobin; MCHC: mean corpuscular hemoglobin concentration; RDW: red cell distribution width; APTT: activated partial thromboplastin time.

Sodium	Latest Ref Range: 135 - 145 mEq/L	140
Potassium	Latest Ref Range: 3.5 - 5.1 mEq/L	3.2 (L)
Chloride	Latest Ref Range: 98 - 107 mEq/L	105
Carbon Dioxide	Latest Ref Range: 22 - 28 mEq/L	15 (L)
Albumin	Latest Ref Range: 3.5 - 5.2 G/dL	4.2
Blood Urea Nitrogen	Latest Ref Range: 8 - 23 mg/dL	17
Creatinine	Latest Ref Range: 0.66 - 1.25 mg/dL	0.8
Calcium	Latest Ref Range: 8.8 - 10.2 mg/dL	8.5 (L)
Lactic Acid	Latest Ref Range: 0.7 - 2.1 mmol/L	6.8 (H)
Magnesium	Latest Ref Range: 1.7 - 2.4 mg/dL	1.9
Phosphorus	Latest Ref Range: 2.5 - 4.5 mg/dL	3.9
Troponin High Sensitive (Dhahran Only)	Latest Ref Range: 0.000 - 0.026 ng/mL	0.073 (HH)
B-type Natriuretic Peptide	Latest Ref Range: <100 pg/mL	1824.00 (H)
Bilirubin, Total	Latest Ref Range: 0.2 - 1.3 mg/dL	1
Alk. Phosphatase	Latest Ref Range: 56 - 119 IU/L	73
ALT (SGPT)	Latest Ref Range: <50 IU/L	26
AST (SGOT)	Latest Ref Range: 17 - 59 IU/L	88 (H)
WBC	Latest Ref Range: 4.0 - 10.0 K/cumm	18.2 (H)
RBC	Latest Ref Range: 4.5 - 5.9 M/cumm	4.59
Hemoglobin	Latest Ref Range: 13.5 - 17.5 g/dL	12.1 (L)
Hematocrit	Latest Ref Range: 41 - 53 %	38.0 (L)
MCV	Latest Ref Range: 77 - 96 fL	82.8
MCH	Latest Ref Range: 26 - 34 pg	26.4
MCHC	Latest Ref Range: 32 - 36 g/dL	31.8 (L)
RDW	Latest Ref Range: 10.9 - 15.7	15.6
Platelets	Latest Ref Range: 150 - 450 K/cumm	276
Mean Platelet Volume	Latest Ref Range: 9.0 - 13.0 fL	10.1
Platelets Distribution Width	Latest Ref Range: 10.1 - 16.1 fL	11.4
Neutrophil %	Latest Units: %	77
Neut. Absolute	Latest Ref Range: 1.8 - 7.0 K/cumm	14.0 (H)
Lymphocyte %	Latest Units: %	10
Lymph. Absolute	Latest Ref Range: 0.9 - 4.9 K/cumm	1.8
Monocyte %	Latest Units: %	2
Mono. Absolute	Latest Ref Range: 0.0 - 1.0 K/cumm	0.3
Eosinophil %	Latest Units: %	0
Eos. Absolute	Latest Ref Range: 0.0 - 0.4 K/cumm	0
Basophil %	Latest Units: %	0
Baso. Absolute	Latest Ref Range: 0.0 - 0.1 K/cumm	0
Band Neutrophil %	Unknown	11
Band Neutrophil Absolute	Unknown	1.98
Poikilocytosis	Unknown	3
Ovalocytes	Unknown	1
Echinocytosis	Unknown	2
Schistocytosis	Unknown	1
Giant Platelet	Unknown	Seen
Prothrombin Time	Latest Ref Range: 9.8 - 12.7 Secs	12.5
I.N. Ratio	Latest Ref Range: 1.0 - 1.2 Ratio	1.2
APTT, Patient	Latest Ref Range: 22 - 33 Secs	26
APTT, Control	Latest Units: Secs	26

EKG revealed sinus tachycardia, possible left atrial enlargement, left ventricular hypertrophy with repolarization abnormality, ST depression, and T-wave inversion in the inferior leads.

**Figure 1:**
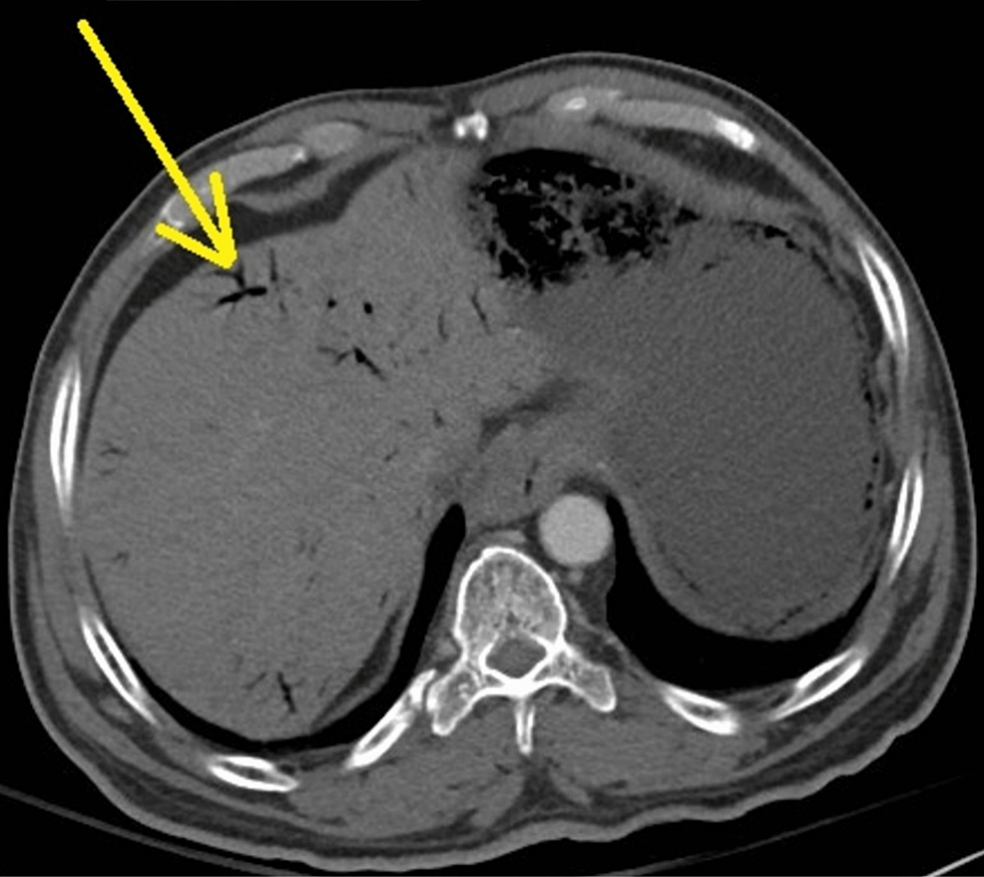
Abdominal CT scan showing portal vein pneumatosis (yellow arrow)

**Figure 2:**
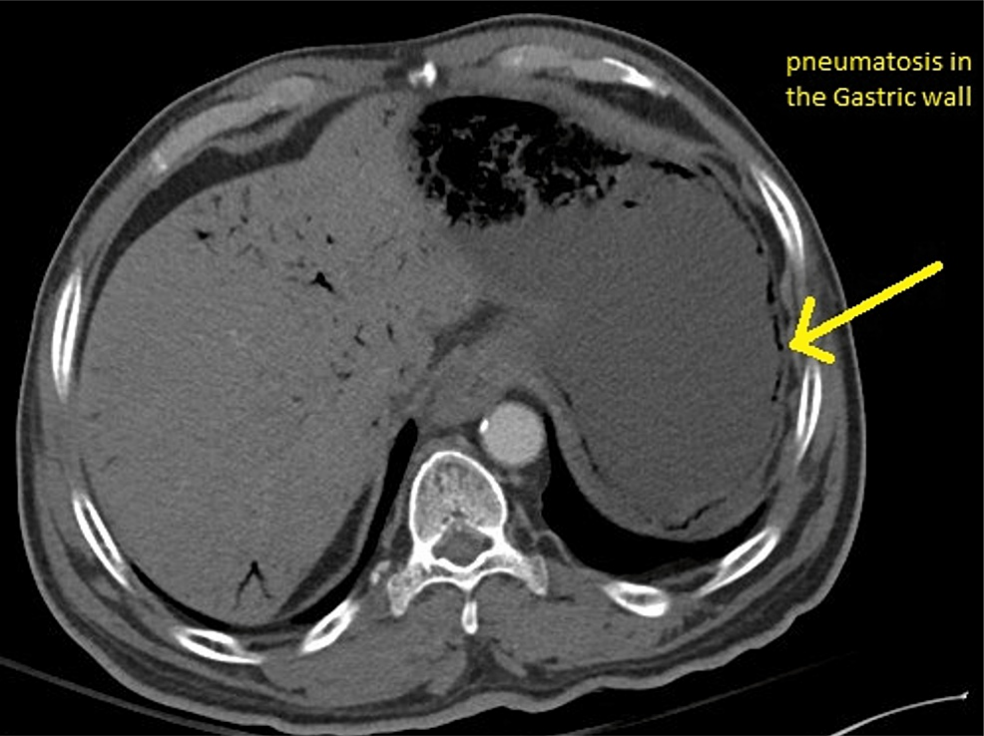
Abdominal CT scan showing gastric wall pneumatosis (yellow arrow)

CT scan of the abdomen was initially done without contrast showing a distended stomach with pneumatosis with portal venous gas, suspected secondary to mesenteric ischemia. Mildly dilated small bowel loops with feces and air-fluid levels and apparent transition in the distal ileum were observed, which may represent small bowel obstruction. The abdominal aorta and its branches demonstrated atheromatous calcification without evidence of aneurysm. There was no free fluid or free air. Repeated CT abdomen with IV contrast, after a few hours, showed extensive pneumatosis of the stomach and poor perfusion of the gastric wall in the vicinity of the gastric fundus. The proximal small bowel was distended and fluid-filled measuring about 3 cm in diameter. The distal small bowel measured about 1.5 cm. There was retained stool in the mid and distal small bowel. Low-grade small bowel obstruction was considered. CT angiography was done showing patent abdominal aorta and inferior vena cava. Diffuse vascular calcifications were also present. The celiac, superior mesenteric artery, and the inferior mesenteric artery were patent. Patent superior mesenteric vein and portal vein were observed. There were no signs of retroperitoneal lymphadenopathy, intra-abdominal free fluid, or free air. CT abdomen showed a distended stomach with extensive pneumatosis and significant intraportal venous air (Figures 1-2). Gastroscopy was considered due to poor enhancement of the gastric fundus to exclude gastric ischemia.

The patient was admitted to the ICU and was started on resuscitation with IV fluids, NPO, broad-spectrum IV antibiotics, analgesia, nasogastric tube (NG), and suction. Oesopghago-gastro-duodenoscopy (OGD) was ordered to check the gastric wall. Diagnostic laparoscopy/laparotomy was considered depending on the clinical picture. 

During the hospital stay, the plan was to continue cautious conservative management with broad-spectrum antibiotics and intravenous proton pump inhibitors as well as good hydration. We planned to follow-up with abdominal X-rays to rule out pneumoperitoneum. Surgery was indicated if there was evidence of perforation or deteriorating clinical condition. Later, the patient showed early signs of deterioration with increased lactic acid levels and was started on ionotropic support to maintain blood pressure. Consent was obtained from the family for diagnostic laparoscopy and possible bowel resection and laparotomy.

Intraoperatively, the patient had OGD carried out at the beginning of surgery which revealed ischemic patches in the gastric mucosa, but the duodenum was intact. Extensive patchy ischemia of both the small and large bowel, affecting 2.5-3 meters of the mid small bowel, was seen. Ischemia of the left colon starting at the distal transverse colon up to the distal sigmoid and dilated stomach was observed.

Diagnostic laparoscopy followed by midline incision laparotomy was done. Resection of the ischemic small bowel along with the left-sided colon was carried out. Resection of the ischemic part of the small bowel was done, sparing about 75 cm of the proximal jejunum from the duodenojejunal junction and 50 cm of the terminal ileum from the iileocecaljunction. The terminal ileum was closed with TLC 75 stapler (Ethicon, Edinburgh, UK). The proximal small bowel was brought out in the left upper quadrant and matured as mucus fistula using 2/0 Vicryl Rapid (Ethicon, Somerville, NJ). The resected ischemic small and large bowel was sent for histology. Mass closure of the midline wound was performed with a polydioxanone (PDS) loop (Ethicon, Somerville, NJ).

Another surgery was planned after 48-72 hours to evaluate for ongoing or newly formed ischemia; however, there was no further evidence of bowel ischemia. The previous ischemic patch in the stomach had improved and became very small on the anterior wall. The right colon was brought out through the right upper quadrant as a mucus fistula was matured with 2/0 Vicryl Rapid. Mass closure of the midline incision was performed with loop PDS x 2.

Pathology showed transmural ischemia of the involved portion of the large and small bowel. Examination of mesenteric vasculature failed to show any emboli. The mesenteric vasculature was evaluated extensively, and any form of vasculitis/thrombus/embolus was ruled out.

Postoperatively, the patient improved and was hemodynamically stable. He came off inotropes, was extubated on the second postoperative day, and started on liquids which he tolerated. The patient continued to be on total parenteral nutrition (TPN) which finally stopped on the 6th postoperative day and the patient commenced his regular diet. During his admission, consultations were sought from cardiology and psychiatry for his agitation, confusion, history of mental health, and substance misuse.

The plan was to re-operate after stabilizing his cardiac condition to reestablish GI continuity during two-stage surgery.

## Discussion

Bowel ischemia is a condition caused by interrupted blood flow to the bowel. Different pathologies can be attributed to bowel ischemia, either occlusive or non-occlusive. Thromboembolism accounts for the majority of occlusive obstruction [9]. On the other hand, non-occlusive obstruction is caused by a number of pathologies. The most common are cardiac failure, arrhythmia, or low-flow states with hypoperfusion [10].

In this case, the CT angiogram of the patient did not show any occlusive cause of bowel ischemia; there were no thrombi or emboli. The patient had diffuse calcification of his vessels but all the major blood vessels including the celiac, superior mesenteric artery, and inferior mesenteric artery were patent. Furthermore, the patient was maintained on oral anticoagulants at the time of admission. The patient was hemodynamically stable; there were no signs or symptoms of heart failure. No arrhythmia was noticed apart from sinus tachycardia. Shortly after admission, the patient deteriorated and was started on ionotropic support to maintain his blood pressure, and this could be attributed to bowel ischemia leading to sepsis and hypotension. Nevertheless, we believe that the septic shock requiring inotropes was secondary to ischemic insult of the small and larger bowel, and there was some non-occlusive cause leading to bowel ischemia which in our case could be due to Illicit methamphetamine use.

Rarely non-occlusive obstruction can be caused by vasoactive drugs; in our case, the patient had a significant history of methamphetamine drug abuse. Methamphetamines have a sympathomimetic effect and cause vasoconstriction in splanchnic blood circulation. It increases the levels of catecholamine by increasing its release and at the same time, by decreasing its uptake at the presynaptic terminal [11]. Excess catecholamine can act on alpha-1 receptors in the arteriolar smooth muscle and produce vasoconstriction which could explain the cause of bowel ischemia in our patient. Methamphetamine can cause mesenteric ischemia or infarction by different mechanisms including, necrotizing vasculitis, cardiovascular shock, sympathomimetic vasospasm, or splanchnic vasoconstriction; it remains unclear how important each of these mechanisms is to induce bowel ischemia. Methamphetamine through vasoconstriction can act upon the mesenteric blood vessels leading to ischemia and bowel necrosis. This can be associated with septic shock and multiorgan failure without intervention. Other adverse effects of methamphetamines include tachycardia, hypertension, arrhythmia, acute coronary syndrome, psychosis, peripheral vasospasm, and dependence [12-14].

In this case, the patient declared methamphetamine illicit drug abuse and was followed by psychiatry for his associated depression and psychosis. No other alcohol or illicit drug abuse was revealed except daily methamphetamine powder (Shabo) till the day of hospital admission and heavy smoking.

The diagnosis of this case was reached by CT angiography which is considered the gold standard for the diagnosis of mesenteric ischemia. Interestingly, we noticed gastric emphysema and portal venous gas with patent mesenteric vessels. CT angiography failed to show occlusive causes, this points to the non-occlusive cause of bowel ischemia. The patient was hemodynamically stable on admission, and this ruled out hypo-perfusion as a cause of bowel ischemia.

Mortality of non-occlusive intestinal ischemia is estimated to be around 70%-90% [15]. In recent years, mortality among people who injected illicit drugs has increased. A systematic review and meta-analysis found that the crude mortality rate of injected illicit drug users is estimated to be 2.35 death per 100 person-years [16]. Methamphetamine abuse is also associated with emotional decision-making, increased impulsivity, and reduced mental flexibility [17]. This poses a challenge in recovery especially in elderly patients as the recovery enables the patient to monitor and inhibit the behavior and facilitate planning and decision making [18].

This interesting case demonstrates that enquiring about stimulant drug use is crucial even for an older patient. Drug abuse is not only for young adults; older persons are also susceptible to it and can present with a unique stimulant-induced surgical, medical, and psychiatric pathology. The use of recreational drugs by elderly populations is largely unrecognized but it is increasing [19]. This medical complication was revealed in the history of our patient. In the longer-term care for the patient, cognitive behavioral interventions have been associated with a reduction in stimulant abuse, and risk-taking behavior, and a reduction in the associated depression and mental illness [20].

## Conclusions

We described a case report of a 67-year-old elderly male patient with a history of methamphetamine illicit drug abuse who presented with acute abdominal pain and bowel ischemia which appeared to be related to methamphetamine abuse. This case aims to raise awareness of bowel ischemia presenting with acute abdominal pain in the unusual context of methamphetamine abuse in elderly patients. This has surgical, medical, psychiatric, and critical care dimensions. A delay in diagnosis and interventions is associated with high morbidity and mortality. Frequent clinical examinations help in early diagnosis and surgical intervention when the CT findings are equivocal in the dynamic assessment process. Surgical along with psychiatric interventions for the treatment of amphetamine dependence is the mainstay of treatment.
